# Regulation of Cell Transformation by Rb-Controlled Redox Homeostasis

**DOI:** 10.1371/journal.pone.0102582

**Published:** 2014-07-14

**Authors:** Zhongling Zhu, Yuanyuan Wang, Zheng Liang, Wenwen Wang, Huamei Zhang, Binghui Li, Guoguang Ying

**Affiliations:** Laboratory of Cancer Cell Biology, Key Laboratory of Breast Cancer Prevention and Therapy, Tianjin Medical University Cancer Institute and Hospital, Tianjin, People’s Republic of China; University of Alabama at Birmingham, United States of America

## Abstract

Rb is a tumor suppressor, and regulates various biological progresses, such as cell proliferation, development, metabolism and cell death. In the current study, we show that Rb knockout in 3T3 cells leads to oxidative redox state and low mitochondrial membrane potential by regulating mitochondrial activity. Our results indicate that Rb plays an important role in controlling redox homeostasis. More importantly, the functions of Rb in modulating cell proliferation, death and transformation are, at least in part, mediated by its controlling cellular redox state. In addition, our results also suggest that the cellular redox state possibly determines various biological activities, including cell survival, death and transformation, where Rb is functioning as a regulator of redox homeostasis.

## Introduction

The retinoblastoma protein (Rb) is a well known tumor suppressor, and functions in the control of cell cycle progression and proliferation [1]. In this context, Rb usually acts as a negative regulator of transcription mediated by the E2F family of transcription factors and inhibits the G1-S phase transition. The function of Rb is modulated through changes in its phosphorylation status, which is mainly conducted by cyclin-dependent kinase (CDK)-cyclin complexes. In addition, Rb has been demonstrated to have many other functions, such as preservation of chromosomal stability, induction and maintenance of senescence, regulation of apoptosis, cellular differentiation and angiogenesis [2]. All these processes play crucial roles in preventing tumor progression, and thus probably also contribute to Rb tumor suppressor function.

Besides the canonical pathways that link Rb tumor suppressor to human cancers, recent studies have shown an essential role for Rb in the regulation of cell metabolism [3]. The Rb-E2F1 complex can translate signals that sense the metabolic needs of the cell into a transcriptional response and orchestrate a complex control of oxidative and glycolytic metabolisms [4]. This is consistent with a notion that cells have to coordinate proliferative and metabolic pathways for growth. Being involved in the regulation of both proliferation and metabolism, Rb appears to play a critical role in such functional integration. Rb inactivation is frequently found in various human cancers [5], and accordingly, cancer cells have many specific metabolic phenotypes, such as glutamine addiction [6], [7] and Warburg Effect, which is a shift of ATP generation pathway from oxidative phosphorylation to glycolysis even under normal oxygen concentrations [8], [9]. At present, there is substantial evidence that loss of Rb function causes an increase in glycolysis, a hallmark of cancer, and facilitates the usage of glutamine for oxidative phosphorylation [3]. In the meantime, Rb has been also shown to regulate redox homeostasis-coupled glutathione (GSH), and loss of Rb leads to a significant change in the GSH/GSSG (oxidized glutathione) balance [10]. Additionally, Rb and E2F can control the accumulation of reactive oxygen species (ROS) and Rb inactivation induces substantial oxidative stress [11]–[13]. Oxidative stress and redox homeostasis are in essence associated with and integrated in metabolism, and thereby, these observations confirm the role of Rb in regulating cellular metabolism.

The changes acquired by cancer cells that cause their unregulated proliferation and growth usually include both oncogenic pathways and inactivated tumor suppressor pathways [14]. Currently, strategies to develop targeted cancer therapies generally aim at components of oncogenic signaling pathways that are deregulated or required in cancer cells, such as specific kinases [15]–[18]. Unfortunately, cancers eventually develop resistance to such therapies [19], [20]. Characterization of the precise metabolic pathways modulated by Rb tumor suppressor should enable the identification of selective therapeutic targets other than current ones involved in oncogenic pathways. At present, some Rb-associated metabolic enzymes, such as lactate dehydrogenase (LDH), glucose transporter 1 (Glut1) and 6-phosphofructo-2-kinase (PFKFB), are suggested to be potential targets for Rb-deficient cancer cells [3]. In addition, based on the fact that Rb controls metabolic stress, a recent report demonstrates that inactivating TSC2 can specifically kill Rb mutant cancer cells by further promoting anabolism to induce cellular stress, indicating a new therapeutic strategy depending on Rb-regulated metabolism [12]. Therefore, dissection of the role of Rb-controlled metabolic homeostasis in tumor progression may allow developing therapies by specifically targeting loss of Rb function in cancer cells.

## Materials and Methods

### Chemicals and reagents

N-acetyl-L-cysteine (NAC), dihydroethidium (DHE), propidium iodide (PI) and hydrogen peroxide (H_2_O_2_) (30%) were obtained from Sigma (USA). ROS dyes H2DCFDA (5-(and-6)-chloromethyl-2′7′-dichlorodihydrofluorescein diacetate acetyl ester), JC-1 (5,5′,6,6′-tetrachloro-1,1′,3,3′-tetraethylbenzimidazolylcarbocyanine iodide) and MitoTracker Red were obtained from Invitrogen (USA). NAC were dissolved in the growth medium. PI was dissolved in water. DHE and JC-1 were dissolved in DMSO as a stock buffer.

### Cell culture

3T3/wt and 3T3/Rb^−/−^ cells were maintained in DMEM (high glucose) supplemented with 10% fetal bovine serum (Hyclone, USA) and 50 IU penicillin/streptomycin (Invitrogen, USA), and MCF-10A cells were cultured in DMEM/F12 containing 5% horse serum (Hyclone, USA), 20 ng/ml EGF (Roche, USA), 0.5 mg/ml hydrocortisone (Sigma, USA), 100 ng/ml cholera toxin (Sigma, USA), 10 µg/ml insulin (Sigma, USA) and 50 IU penicillin/streptomycin (Invitrogen, USA) in a humidified atmosphere with 5% CO_2_ at 37°C.

### Soft agar growth assay

For soft agar assay, 10^4^ cells suspended in top agarose solution (0.3%) were poured over bottomed agarose (0.6%) previously solidified in 6-well plates. Cells were cultured in a humidified atmosphere with 5% CO_2_ at 37°C for weeks, and then colonies were counted.

### FACS analysis

For the apoptosis assay, 25×10^4^ cells were seeded into each well of the 6-well plates. Apoptosis assays were carried out based on the instruction from the Annexin V Apoptosis Kit (BD Biosciences). Briefly, cells were collected and washed twice with binding buffer containing 10 mM HEPES, pH 7.4, 140 mM NaCl, 2.5 mM CaCl, and then resuspended at a concentration of 1×10^6^ cells/ml in binding buffer. Hundred microliters of the cell suspension was mixed with 5 µL of Annexin V-FITC (BD Biosciences) and 10 µL of propidium iodide (50 lg/ml stock) and incubated at room temperature for 15 min. Four hundred micro liters of binding buffer was added to each assay after the incubation and apoptotic cells were determined using a FACScan (BD Biosciences). Since NAC or H_2_O_2_-induced cell death in 3T3 cells is mostly apoptosis, total Annexin V-positive cells were used to determine the level of apoptosis.

Intracellular ROS production was monitored by the permeable fluorescence dye, H2DCFDA and/or DHE. H2DCFDA can readily react with ROS to form the fluorescent product 2,7-dichlo-rofluorescein (DCF) [21]. DHE oxidation is particularly sensitive to O_2_^−^ and hydroxyl radicals [22]. The intracellular fluorescence intensity of DCF or DHE is proportional to the amount of ROS generated by the cells [23]. After the indicated treatment, the cells were incubated with 10 µM of H2DCFDA or DHE dissolved in PBS for thirty minutes and then cells were harvested and resuspended in PBS (10^6^ cells/ml). The fluorescence intensity of intracellular DCF (excitation 488 nm, emission 530 nm) or DHE (excitation 518 nm, emission 605 nm) was measured using FACScan (BD Biosciences, USA).

The mitochondrial membrane potential (MMP) was monitored by the MMP probe JC-1 dye, which emits red florescence (590 nm, FL2-H, Red/Orange) under high MMP and green florescence (525 nm) under low MMP conditions [24]. The MMP assay was carried out according to the previous report [25] with a few modifications. Briefly, after desired treatments, cells were collected, resuspended in complete medium containing 2.5 µM of completely dissolved JC-1. The mixture was incubated at 37°C, 5% CO_2_ for 30 min. Cells were collected, resuspended in 0.5 ml of PBS for Flow assay. All the data analyses were performed using FlowJo analysis software, version 6.0 (Tree Star).

### Imaging of cultured cells

After cells were grown in 6-well plates for 24 h, they were cultured in the complete medium containing 500 nM of MitoTracker Red for 30 min, at 37°C, 5% CO_2_, washed twice with warm medium, and then immediately analyzed under a Nikon Eclipse TE2000-U fluorescence microscope (Nikon, Japan). The fluorescence intensity of images was analyzed with ImageJ software (1.47).

### Measurements of redox state

We used genetically-encoded Grx1-roGFP2 fusion protein to measure the redox state in cells [26]. Grx1-roGFP2 protein allowed dynamic live measuring of the glutathione redox potential, and it has two fluorescence excitation maxima at about 400 and 490 nm and show rapid and reversible ratiomertic changes in fluorescence in response to changes in redox potential. Grx1-roGFP was stably expressed in cells using lentivirus. After desired treatments, cells were imaged with a Nikon Elicipse TE2000-U fluorescence microscope (Nikon, Japan). Dual-excitation ratio imaging used excitation filters 400DF10 and 480DF15. A 505DRLP dichroic mirror and an emission filter, 535DF20, were used for both excitations. Raw data were exported to ImageJ software (1.47) as 16-bit TIF for analysis. Background correction was performed by subtracting the intensity of a nearby cell-free region from the signal of the imaged cell and a threshold was set to avoid ratio-created artifacts. Fluorescence excitation ratios were obtained from manually selected portions of intact whole cells in the ratio images by dividing the 400 nm picture by the 480 nm image pixel by pixel. The 400/480 ratio value for each treatment was the mean of ratios obtained from of 20 cells. Pseudocolor ratio pictures were created by using the ImageJ look-up table ‘Fire’.

The ratios of GSSG/GSH and NADP+/NADPH in 3T3 cells were measured using Glutathione (GSH/GSSG) Fluorometric Assay Kit and NADP/NADPH Quantitation Colorimetric Kit (BioVision, USA) according to the manual instruction.

### Cell cycle analysis

Cells were seeded in 10-cm dishes for 48 h, trypsinized to single cells, and then washed twice with PBS. 1 ml of cells (about 10^6^ cells) was aliquoted in a 15 ml polypropylene tube and 3 ml of cold absolute ethanol was added dropwise while vortexing. Cells were fixed overnight at 4°C, washed twice with PBS, and then incubated with 1 ml of PI staining buffer (10 mM Tris pH 7.5, 5 mM MgCl2, 20 µg/ml RNase A and 5 µg/ml PI) for 30 minutes at 37°C. Cells were analyzed using a FACScan (BD Biosciences, USA). Cell cycle distribution was analyzed using FlowJo (6.0) software (Tree Star).

### RNA isolation and RT-PCR

Cells were cultured and treated as indicated in the content and then collected. Total RNA was extracted with a TRIzol procedure as specified by the manufacturer (Invitrogen, USA). Purified RNA was quantified by spectrophotometry. The mRNA levels of Bax, Bak, Bad and GAPDH were detected by standard RT-PCR (reverse transcription system and the expand high fidelity PCR system (Thermo, USA)) using a random primer and then the specific primers as following:

Mouse GAPDH-F: gcacagtcaaggccgagaat
Mouse GAPDH-R: gccttctccatggtggtgaa
Mouse Bax-F: aggatggctggggagacacc
Mouse Bax-R: ctgccacccggaagaagacc
Mouse Bak-F: cgctacgacacagagttcca
Mouse Bak-R: ggtagacgtacagggccaga
Mouse Bad-F: gcttagcccttttcgaggac
Mouse Bad-R: cccaccaggactggataatg

GAPDH mRNA level was used as the normalization control.

### Western blot

After desired treatments as specified as indicated, cells were washed twice with PBS and lysed in buffer (20 mM Tris-HCl, pH 7.5, 150 mM NaCl, 1 mM EDTA, 1% Triton X-100, 2.5 mM sodium pyrophosphate, 1 mM β-glycerophosphate, 1 mM sodium vanadate, 1 mg/ml leupeptin, 1 mM phenylmethylsulfonylfluoride). Equal amounts of protein (30 µg) were loaded onto 15% SDS-PAGE gels. Western detection was carried out using a Li-Cor Odyssey image reader (Li-Cor, USA). Anti-Rb, anti-Bad, anti-myc, anti-Ras and anti-β-Actin antibodies were obtained from Cell Signaling Technology (CST, USA), and were used with a dilution of 1∶1000. Anti-Bax, anti-Bak and anti-Bcl-XL antibodies were from Bioss (China), and were diluted by 1∶100. The goat anti-mouse immunoglobulin G (IgG) and goat anti-rabbit IgG secondary antibodies were obtained from Li-Cor (USA). The final concentration of the secondary antibodies used was 0.1 µg/ml.

### Lentivirus production

Mitochondrial target sequence of human pyruvate dehydrogenase alpha 1 [27] was used to construct mitochondria-targeted catalase. The sequence was shown here: ATGAGGAAGATGCTCGCCGCCGTCTCCCGCGTGCTGTCTGGCGCTTCTCAGAAGCCGGCAAGCAGAGTGCTGGTAGCATCCCGTAATTTTGCAAATGATGCTACATTTThe cDNA of Grx1-roGFP2, a redox sensitive fluorescent probe, was commercially synthesized (Genewiz, China) according to the previous report [26]. All cDNAs for Grx1-roGFP2, Bcl-XL, myc-catalase, myc-Rb, H-Ras (V12 mutant), Gpx1, and GSR were cloned into lentiviral expression vectors, pCDH-puro-CMV or pCDH-Neo-CMV using the eFusion Recombinant Cloning Kit (Biophay, China). The pLKO.1 lentiviral RNAi expression system was used to construct lentiviral shRNA for Rb. The sequences of shRNA used in this study included the following:

shScramble: CCGGCCTAAGGTTAAGTCGCCCTCGCTCGAGCGAGGGCGACTTAACCTTAGGTTTTT
shRb [12]: CCGGCGACGAGTCAAACAAGCCAATCTCGAGATTGGCTTGTTTGACTCGTCGTTTTT

Viral packaging was done according to a previously described protocol [28]. Briefly, expression plasmids pCDH-CMV-cDNA or pLKO.1-shRNA, pCMV-dR8.91, and pCMV-VSV-G were co-transfected into 293T cells using the calcium phosphate coprecipitation at 20∶10∶10 µg (for a 10-cm dish). The transfection medium containing calcium phosphate and plasmid mixture was replaced with fresh complete medium after incubation for 5 h. Media containing virus was collected 48 h after transfection and then concentrated using Virus Concentrator Kit (Biophay, China). The virus was resuspended in the appropriate amount of complete growth medium and stored at −80°C. Cancer cells were infected with the viruses at the titer of 100% infection in the presence of polybrene (10 µg/ml) for 48 h, and then cells were selected with puromycin or neomycin.

### Statistical analysis

Unless otherwise indicated, at least three independent assays were carried out and the values are presented as mean ± S.D. Statistical significance was assessed by Student’s two-tailed t test and *P*<0.05 was considered as statistically significant.

## Results

### Rb controls redox homeostasis

**Figure 1:**
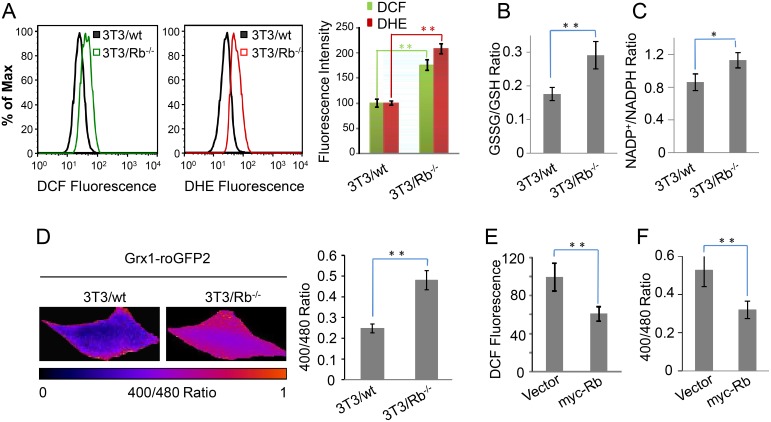
Rb regulates oxidative stress in 3T3 cells. (A) ROS levels were determined based on DCF or DHE fluorescence after cells were cultured for 24 h. (B and C) GSSG/GSH and NADP+/NADPH ratios were measured under the normal culture condition. (D) Cells expressing Grx1-roGFP2 were excited with 400 and 480 nm wavelengths, and the ratio of emission in the channel (535 nm) was calculated (right). Pseudocolor image was shown with the color scale (left). (E) ROS levels were determined based on DCF fluorescence after 3T3/Rb^−/−^ cells with or without Rb re-expression were cultured for 24 h. (F) Ratios of fluorescence obtained at 400/480 nm excitation wavelengths were measured based on JC-1 fluorescence after 3T3/Rb^−/−^ cells with or without Rb re-expression were cultured for 24 h. *P<0.05, **P<0.01.

Rb was implicated in the control of redox homeostasis in *Drosophila* [10]. Here, we used mouse 3T3/wt and 3T3/Rb^−/−^ (Rb knockout) cells to investigate the involvement of Rb in the redox homeostasis. First, we used fluorescent dyes, H2DCFDA that mainly detects the hydroxyl radical [21], and DHE mainly detecting superoxide [22], to determine if Rb deletion induced redox imbalance. Highly elevated fluorescent signals were observed in 3T3/Rb^−/−^ cells compared to 3T3/wt cells (Fig. 1A). Moreover, redox-coupled GSSG/GSH and NADP^+^/NADPH ratios were also increased in Rb knockout cells (Figure 1B and 1C). Therefore, Rb deletion induced significant level of oxidative stress in 3T3 cells. We also quantified the ATP content in both 3T3/wt and 3T3/Rb^−/−^ cell lines, and no difference was observed (data not shown).

To further measure in real-time the redox state of 3T3 cells, we used a genetically-encoded redox-sensitive fluorescent biosensor, Grx-roGFP2, that has two fluorescence excitation maxima at about 400 and 480 nm and displays rapid and reversible ratiometric changes in fluorescence in response to redox changes based on the ratio of GS-SG/GSH in cells [26], [27]. The excitation ratio of 400/480 nm was significantly increased in 3T3/Rb^−/−^ cells (Fig. 1D), indicating an oxidative state, consistent with the results in Fig. 1B. These results show that Rb plays an important role in controlling redox homeostasis. Furthermore, we re-expressed Rb in 3T3/Rb^−/−^ cells and observed a decrease in both ROS level and the excitation ratio of 400/480 nm (Fig. 1E and 1F), confirming the association of Rb with redox homeostasis.

### Rb regulates mitochondrial potential

**Figure 2:**
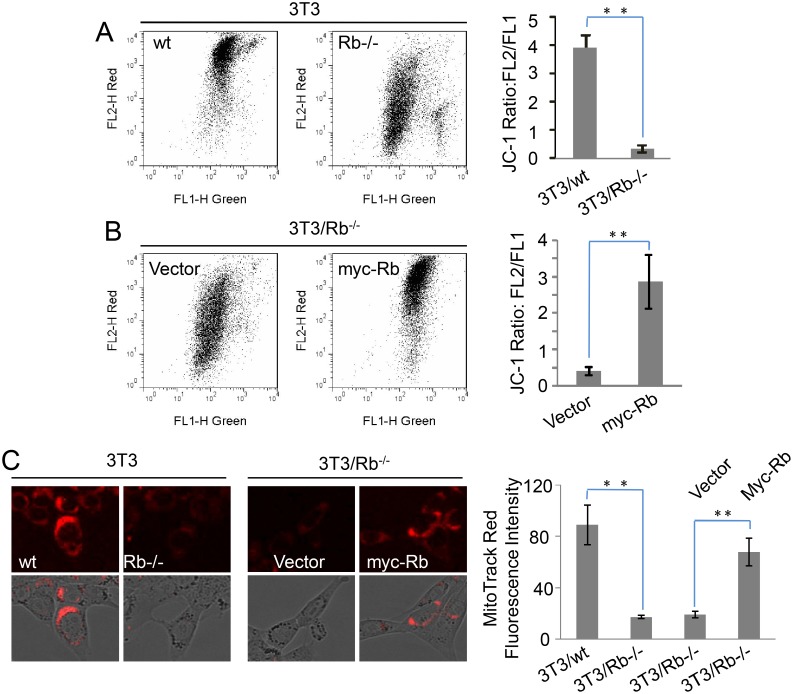
Rb regulates mitochondrial membrane potential. (A) Mitochondrial membrane potential (MMP) was determined by the JC-1 probe. Red fluorescence (FL2) indicates high MMP, while green fluorescence (FL1) indicates low MMP. FL2/FL1 ratio indicates MMP. (B) FL2/FL1 ratios were determined based on JC-1 fluorescence after 3T3/Rb^−/−^ cells with or without Rb re-expression were cultured for 24 h. (C) MMP was determined by MitoTracker Red staining. Red fluorescence indicates high MMP. **P<0.01.

Mitochondria are highly associated with ROS and redox state [29], and thus here we investigated the role of Rb in maintaining the mitochondrial potential in 3T3 cells using JC-1, a dye that either accumulates as J-aggregates in the mitochondria with high membrane potential to become fluorescent red or remains in the monomeric form to show green fluorescence given the low membrane potential [24]. Therefore, the ratio of red fluorescence/green fluorescence (FL2/FL1) reflects mitochondrial membrane potential (MMP). Surprisingly, we observed a significant decrease in the fluorescence ratio of 3T3/Rb^−/−^ cells in contrast to the wild type control cells (Fig. 2A). This suggests that Rb deletion can substantially decrease MMP of 3T3 cells. Furthermore, our data showed that the re-expression of Rb in 3T3/Rb^−/−^ cells apparently increased the FL2/FL1 ratio (Fig. 2B), indicating the important role of Rb in the regulation of MMP. This was further confirmed by the results using another fluorescent dye MitoTracker Red that indicates high MMP with red fluorescence. The fluorescence intensity in wt cells was much stronger than that in Rb knockout cells, and Rb re-expression significantly restored the fluorescence intensity in Rb knockout cells (Fig. 2C). Therefore, Rb is critical in maintaining MMP of 3T3 cells.

**Figure 3:**
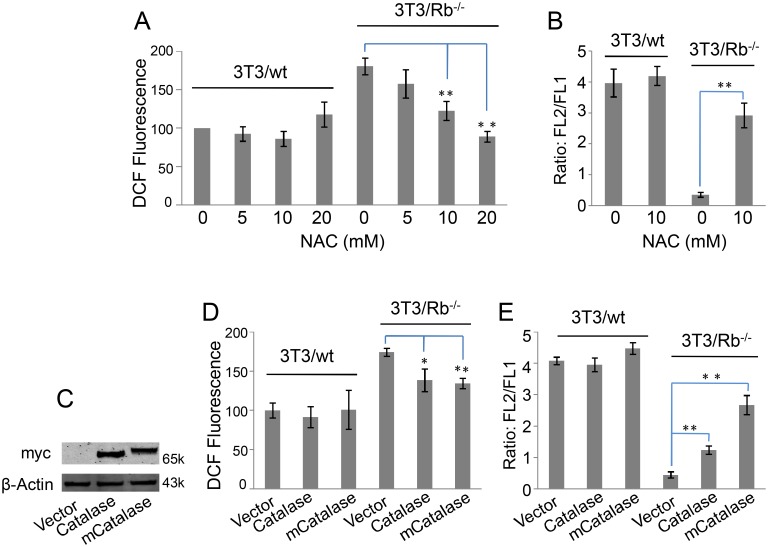
Elevated level of ROS leads to low MMP in 3T3/Rb^−/−^ cells. (A) ROS levels were determined based on DCF fluorescence after 3T3/wt cells or 3T3/Rb^−/−^ cells were treated with different concentrations of NAC for 24 h. (B) FL2/FL1 ratios were determined based on JC-1 fluorescence after 3T3 cells were cultured for 24 h in the presence or absence of 10 mM of NAC. (C) Over-expression of myc-tagged catalase or mitochondria-targeted catalase (mCatalase) was used to scavenge cytoplasmic or mitochondrial ROS. (D) ROS levels were determined based on DCF fluorescence after 3T3 cells with or without catalase/mCatalase expression were cultured for 24 h. (E) FL2/FL1 ratios were determined based on JC-1 fluorescence after 3T3 cells with or without catalase/mCatalase expression were cultured for 24 h. *P<0.05, **P<0.01.

To determine whether the elevated ROS damaged MMP, we used N-acetyl cysteine (NAC), an antioxidant, to reduce oxidative stress. Addition of NAC obviously reduced ROS levels (Fig. 3A) and increased MMP (Fig. 3B) in 3T3/Rb^−/−^ cells while it showed no effect on those in wt cells, suggesting the contribution of elevated ROS to low MMP. The similar results were also obtained from the expression of a ROS scavenging enzyme, catalase. We expressed catalase and mitochondria-targeted catalase (mCatlase) in 3T3 cells (Fig. 3C), and the results showed that both catalases decreased ROS level and maintained higher MMP in Rb knockout cells (Fig. 3D and 3E). Meantime, we noticed that mCatalase was much more effective in restoring MMP than cytoplasmic catalase (Fig. 3E), and therefore mitochondria could be the ROS source.

**Figure 4:**
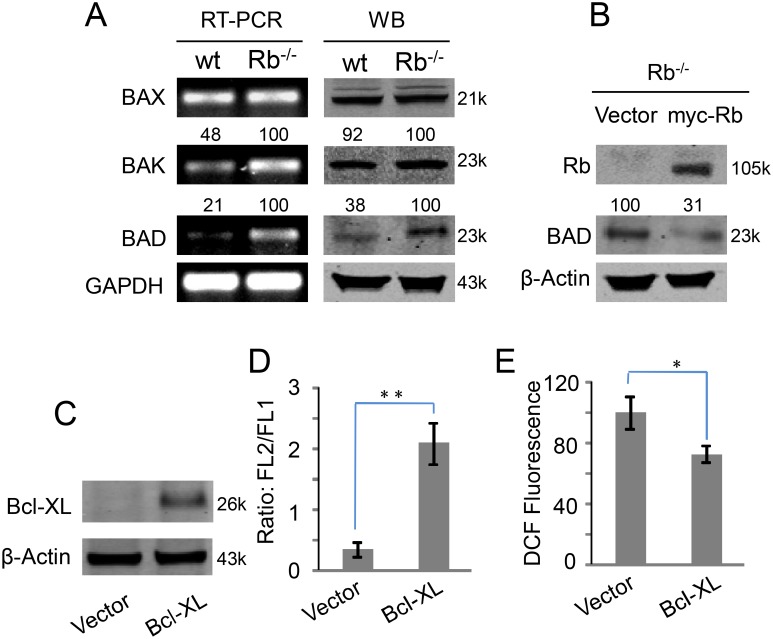
Rb regulates mitochondrial membrane proteins expression. (A) mRNA and protein levels of Bax, Bak and Bad in 3T3/wt and 3T3/Rb^−/−^ cells were determined by RT-PCR and western blot. The number indicates quantified ratio relative to the inter-control GAPDH or β-Actin. (B) Bad protein levels in 3T3/Rb^−/−^ cells with or without Rb re-expression. The number indicates quantified ratio relative to the inter-control β-Actin. (C) Bcl-XL was over-expressed in 3T3/Rb^−/−^ cells. (D) FL2/FL1 ratios were determined based on JC-1 fluorescence after 3T3/Rb^−/−^ cells with or without Bcl-XL over-expression were cultured for 24 h. (E) ROS levels were determined based on DCF fluorescence after 3T3/Rb^−/−^ cells with or without Bcl-XL over-expression were cultured for 24 h. *P<0.05, **P<0.01.

Besides cell cycle, Rb/E2F pathway can control the expression of some BH3-only proteins, such as Bax, Bak and Bad, that are related to mitochondrial activity [30]. Therefore, we tested the expressions of these proteins in 3T3/wt and 3T3/Rb^−/−^ cells. RT-PCR results showed that mRNA levels of Bak and Bad, in particular Bad, were significantly enhanced in 3T3/Rb^−/−^ cells compared to the wt cells (Fig. 4A). Accordingly, the protein level of Bad but not Bak was also increased in 3T3/Rb^−/−^ cells (Fig. 4A). Furthermore, we observed a decrease in Bad protein level in 3T3/Rb^−/−^ cells when Rb was re-expressed (Fig. 4B). These results show that Rb regulates the expression of mitochondrial protein Bad.

Bad can form an inactivating dimer with Bcl-XL and thus increase the permeability of mitochondrial membrane [31], which could lead to reduced MMP and elevated ROS leaking from mitochondrial respiratory chain. Moreover, our forenamed results (Fig. 3E) implied the possibility of mitochondria as the ROS source in Rb knockout cells. Therefore, to further determine if Rb-deletion-induced ROS was initiated from mitochondria in 3T3/Rb^−/−^ cells, we expressed Bcl-XL in these cells (Fig. 4C). Bcl-XL expression significantly increased the MMP (Fig. 4D) and meantime decreased ROS level (Fig. 4E). These data show that Rb regulates redox homeostasis mainly by controlling the mitochondrial activity.

### Rb regulates cell cycle via ROS

**Figure 5:**
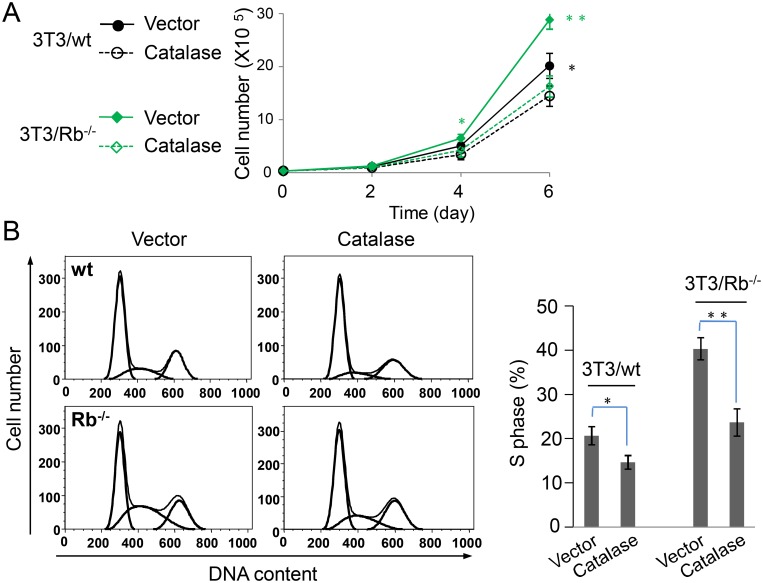
Scavenging ROS inhibits cell growth in 3T3 cells. (A) Cell growth in 3T3/wt and 3T3/Rb^−/−^ cells with or without catalase over-expression was measured. (B) Cell cycle in 3T3/wt and 3T3/Rb^−/−^ cells with or without catalase over-expression was measured. The fraction of cells in S phase from flow data was shown in the right. *P<0.05, **P<0.01.

ROS is implicated in the regulation of cell growth and it either inhibits or promotes cell growth [32]. To investigate the effect of ROS on 3T3 cell growth, we over-expressed catalase in both 3T3/wt and 3T3/Rb^−/−^ cells (Fig. 3C). Catalase expression significantly repressed the growth of Rb knockout 3T3 cells while it also exerted a slightly suppressive effect on wt cells at day 6 (Fig. 5A). Accordingly, it also decreased cell number in S phase of cell cycle, with a much more dramatic decrease in 3T3/Rb^−/−^ cells (Fig. 5B). Rb is well-characterized to control cell cycle and growth. 3T3/Rb^−/−^ cells grew faster with an increased S phase compared to 3T3/wt cells (Fig. 5), indicating that Rb-deficiency-promoted cell growth was possibly mediated by the deregulated cell cycle. Considering that Rb deficiency leads to an elevated level of ROS (Fig. 1A) and that ROS scavenging obviously reduced the fraction of cells in S phase (Fig. 5B), Rb-associated ROS most likely contributed to its cell cycle regulation.

### Rb state determines apoptotic sensitivity of 3T3 cells to redox perturbation

**Figure 6:**
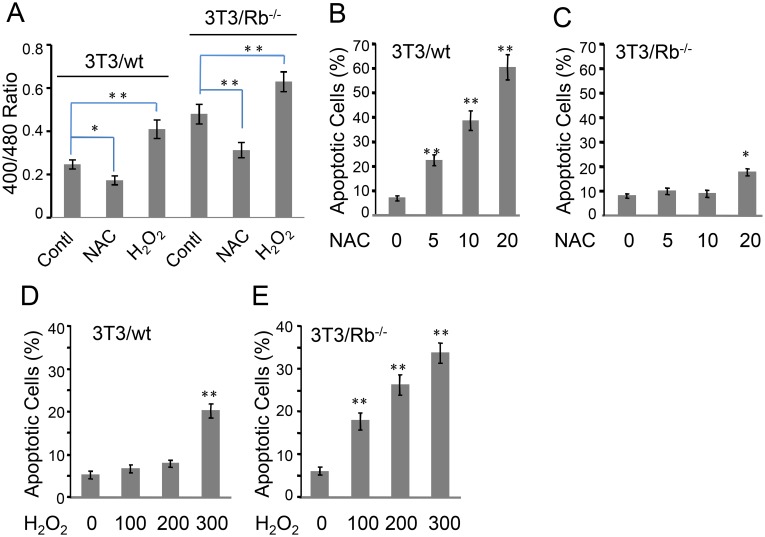
3T3/wt and 3T3/Rb^−/−^ cells show differential response to redox imbalance-induced apoptosis. (A) Ratios of fluorescence obtained at 400/480 nm excitation wavelengths in cells expressing Grx1-roGFP2 were measured in the presence or absence of 10 mM of NAC or 200 µM of H_2_O_2_ for 24 h. (B and C) Apoptosis was determined after 3T3/wt cells (B) or 3T3/Rb^−/−^ cells (C) were treated with different concentrations of NAC for 48 h. (D and E) Apoptosis was determined after 3T3/wt cells (D) or 3T3/Rb^−/−^ cells (E) were treated with different concentrations of H_2_O_2_ for 48 h. *P<0.05, **P<0.01.

We further investigated the effect of redox state on apoptosis in 3T3/wt and 3T3/Rb^−/−^ cells. We used NAC and H_2_O_2_ to adjust the redox state in cells. Although NAC showed a dose-dependent suppression on ROS level in 3T3/Rb^−/−^ cells, it did not further reduce ROS level in 3T3/wt cells (Fig. 3A). This suggested that the basal ROS was actually very low in wt cells. In contrast, the basal ROS level in 3T3/Rb^−/−^ cells was increased due to the lack of Rb, so that NAC exerted the effect of ROS scavenging. In the meantime, we observed that NAC was able to reduce ROS in 3T3/Rb^−/−^ cells to the level in wt cells at most (Fig. 3A) and increasing concentrations of NAC failed to further decrease ROS level in 3T3/Rb^−/−^ cells (data not shown). These observations suggest that the DCF fluorescence intensity in wt cells indeed represents the baseline. However, NAC apparently decreased the excitation ratio of 400/480 nm in wt cells like in Rb knockout cells (Fig. 6A), indicating that NAC still further induced reductive state below the baseline (over-reductive state) in wt cells even if these cells did not have basal ROS. This possibly resulted from the fact that NAC was a cysteine derivative and could act as the precursor of glutathione. It also suggests that the alteration of redox state is not always associated with ROS change. Interestingly, NAC induced apoptosis in 3T3/wt cells in a concentration-dependent manner (Fig. 6B), in sharp contrast, it almost left 3T3/Rb^−/−^ cells unaffected and only slightly increased apoptotic cells upon the treatment with a high concentration of NAC (20 mM) were observed (Fig. 6C). As expected, H_2_O_2_ increased ROS level in both wt and knockout cells (data not shown), and it also induced a more oxidative state in these cells (Fig. 6A). However, 3T3/Rb^−/−^ cells were much more susceptible to H_2_O_2_-induced apoptosis, as compared to 3T3/wt cells (Fig. 6D and 6E), indicating that the further increase in oxidative stress, an over-oxidative state above the level in 3T3/Rb^−/−^ cells, was toxic to these cells that already had a higher level of basal ROS and a more oxidative state than wt cells. Taken all together, these data suggest that 3T3 cells were tolerable towards a fluctuating redox state between the basal levels of wt and 3T3/Rb^−/−^ cells and that the over-reductive or over-oxidative redox state could cause apoptosis in 3T3 cells. In other word, Rb state determined the redox state that predicted the sensitivity of 3T3 cells to the redox perturbation.

### Rb knockout transforms 3T3 cells with an appropriate redox state

**Figure 7:**
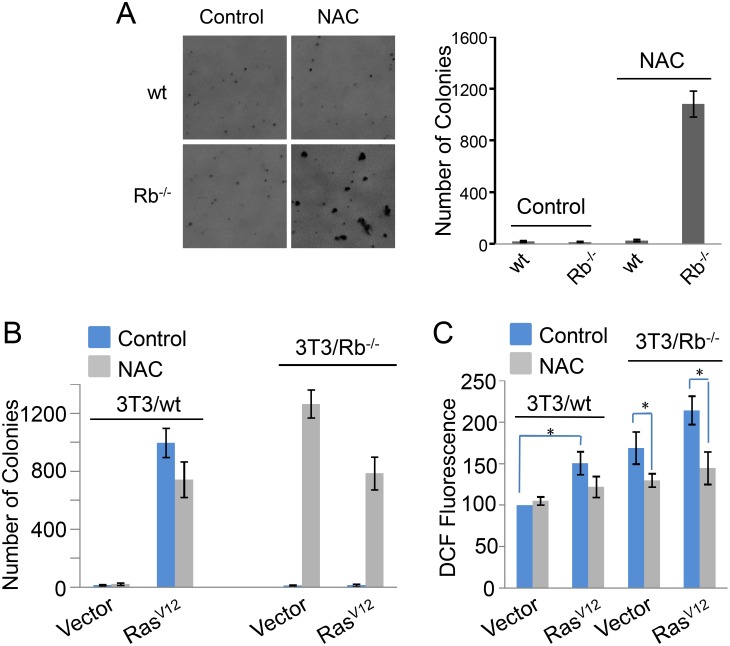
Rb knockout transforms 3T3 cells in the presence of NAC. (A) Colony formation ability of 3T3/wt and 3T3/Rb^−/−^ cells in soft agar in the presence or absence of 10 mM of NAC. (B) Colony formation ability of 3T3/wt and 3T3/Rb^−/−^ cells with or without Ras^V12^ expression in soft agar in the presence or absence of 10 mM of NAC. (C) ROS levels were determined based on DCF fluorescence in 3T3/wt cells or 3T3/Rb^−/−^ cells with or without Ras^V12^ expression in the presence or absence of 10 mM of NAC. *P<0.05.

Rb is characterized as a tumor suppressor, and Rb deletion indeed promotes cell growth in 3T3 cells (Fig. 5). Therefore, we are wondering if Rb deletion can promote the transformation of 3T3 cells. We used soft agar assay to test the colony formation ability of 3T3/wt and 3T3/Rb^−/−^ cells. Our results showed that both wt and Rb knockout cell lines hardly formed colonies in soft agar (Fig. 7A). Interestingly, NAC treatment dramatically increased the soft agar growth of 3T3/Rb^−/−^ cells but not 3T3/wt cells (Fig. 7A).

The constitutively active isoform of Ras, Ras^V12^, can transform 3T3 cells, and meantime produces large amounts of ROS [33]. Our results confirmed that 3T3 cells expressing Ras^V12^ acquired strong ability to grow in soft agar and displayed higher level of ROS than 3T3/Vector cells (Fig. 7B and 7C). However, Ras^V12^ did not induce the colony formation of 3T3/Rb^−/−^ cells in soft agar (Fig. 7B), and actually it was toxic to 3T3/Rb^−/−^ cells (data not shown), consistent with a previous report [34]. Like H_2_O_2_, Ras^V12^ over-expression further increased the oxidative state in 3T3/Rb^−/−^ cells (Fig. 7C), and thus we hypothesized that the “over-oxidative state” might block the transformation ability of Ras^V12^ in 3T3/Rb^−/−^ cells. To test this idea, NAC was used to attenuate the “over-oxidative stress” (Fig. 7C). We found that NAC significantly increased the cell growth of 3T3/Rb^−/−^ cells expressing Ras^V12^ in soft agar (Fig. 7B). These observations strongly support the idea that the radox state determines the transformation outcome of 3T3 cells.

### Redox state not ROS is required for cell transformation

**Figure 8:**
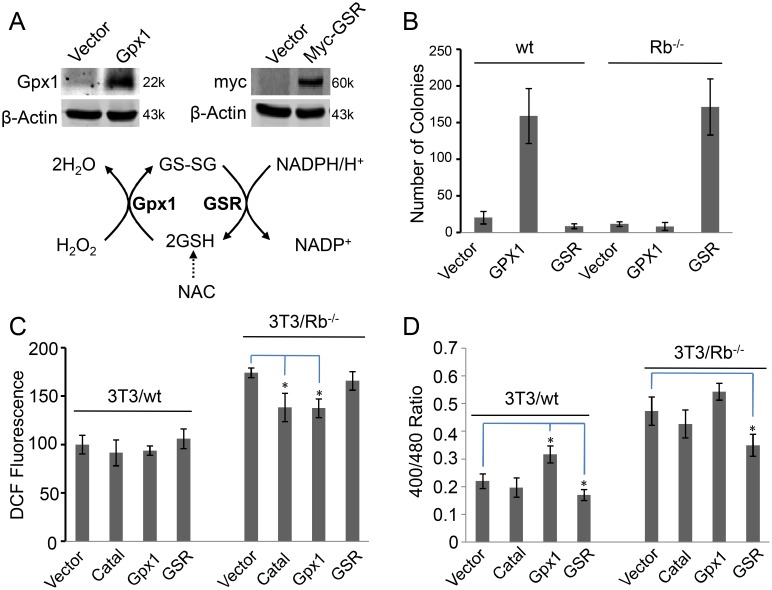
Redox state, not ROS, is corresponding to the transformation ability of 3T3 cells. (A) Glutathione system was artificially modulated by NAC, over-expression of Gpx1 (glutathione peroxidase 1) or GSR (glutathione reductase). (B) Colony formation ability of 3T3/wt cells expressing Gpx1 or GSR and 3T3/Rb^−/−^ cells expressing Gpx1 or GSR. (C) ROS levels were determined based on DCF fluorescence in 3T3/wt cells expressing catalase, Gpx1 or GSR and 3T3/Rb^−/−^ cells expressing catalase, Gpx1 or GSR. (D) Ratios of fluorescence obtained at 400/480 nm excitation wavelengths in 3T3/wt cells expressing catalase, Gpx1 or GSR and 3T3/Rb^−/−^ cells expressing catalase, Gpx1 or GSR. *P<0.05, **P<0.01.

ROS is an important indicator of redox imbalance, and we therefore further investigated the effect of a ROS scavenger enzyme, catalase, on the transformation of 3T3/wt and 3T3/Rb^−/−^ cells. Intriguingly, our results showed that expression of catalase did not show any increase in the cell growth of 3T3/wt or 3T3/Rb^−/−^ cells in soft agar (data not shown), unlike antioxidant NAC that promoted colony formation of 3T3/Rb^−/−^ cells (Fig. 7A). NAC is the precursor of glutathione, and thus it scavenges ROS mainly by stimulating the glutathione system that is associated with redox homeostasis [35], as shown in Fig. 8A. In contrast, catalase directly removes ROS. Our results in Fig. 3A and 6A showed that the change in redox state was not always corresponding to ROS alteration. Therefore, to determine whether ROS or redox state is required for the transformation of 3T3 cells, we expressed Gpx1 (glutathione peroxidase 1) or GSR (glutathione reductase) in 3T3/wt cells or 3T3/Rb^−/−^ cells to adjust the glutathione redox homeostasis (Fig. 8A). Expression of Gpx1 in 3T3/wt or expression of GSR in 3T3/Rb^−/−^ cells significantly increased their colony formation ability in soft agar (Fig. 8B). On the contrary, expression of GSR in 3T3/wt cells or expression of Gpx1 in 3T3/Rb^−/−^ cells failed to promote their soft agar growth (Fig. 8B). In the meantime, we observed the distinct effects of catalase, Gpx1 and GSR on ROS level and redox state (Fig. 8C and 8D). None of catalase, Gpx1 and GSR could further reduce ROS level in wt cells (Fig. 8C), because wt cells already had very low basal ROS level (Fig. 3A). In contrast, only Gpx1 promoted a relatively oxidative redox state in 3T3/wt cells, accompanying an increase in soft agar growth (Fig. 8B and 8D). Catalase and Gpx1 slightly decreased ROS level in Rb knockout cells but only GSR expression that enhanced the growth of 3T3/Rb^−/−^ cells in soft agar attenuated their oxidative redox state measured by Grx1-roGFP. These data suggest that the proper redox state but not ROS is the critical factor affecting the transformation ability of 3T3 cells.

### Redox homeostasis is critical to the transformation of MCF-10A cells

**Figure 9:**
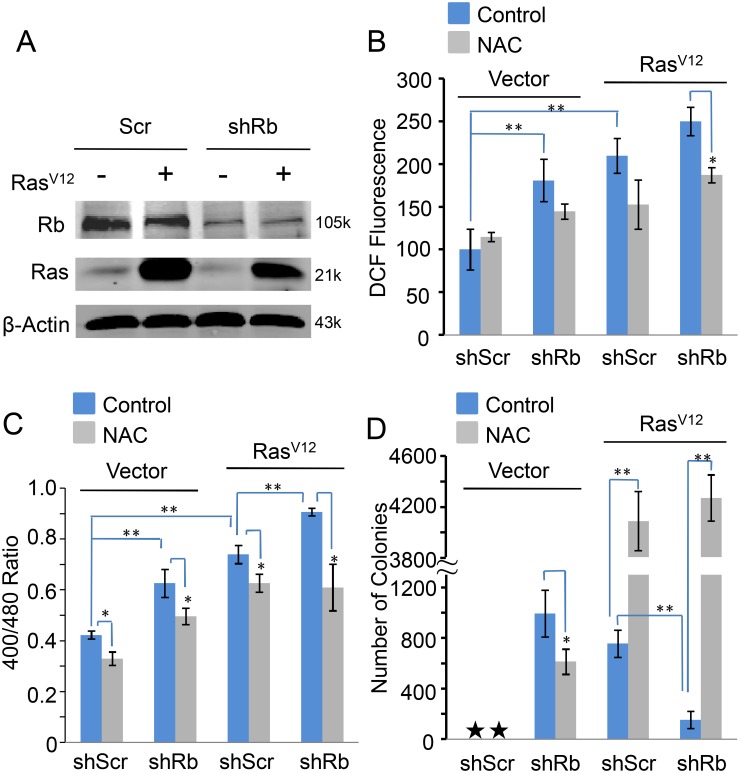
Redox state is critical to the transformation of MCF-10A cells. (A) Over-expression of Ras^V12^ and/or knockdown of Rb in MCF-10A cells. (B) ROS levels were determined based on DCF fluorescence in MCF-10A cells expressing Ras^V12^ and/or shRb in the presence or absence of 10 mM of NAC. (C) Ratios of fluorescence obtained at 400/480 nm excitation wavelengths in MCF-10A cells expressing Ras^V12^ and/or shRb in the presence or absence of 10 mM of NAC. (D) Colony formation ability of MCF-10A cells expressing Ras^V12^ and/or shRb in the presence or absence of 10 mM of NAC. *P<0.05, **P<0.01.

**Figure 10:**
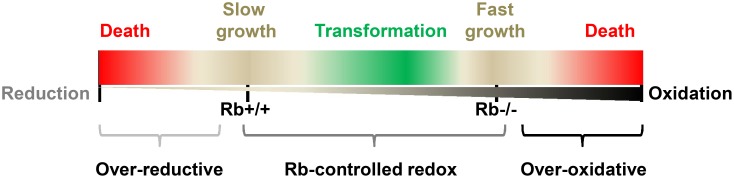
A summary scheme for the effect of Rb-controlled redox on cell fate. Different redox states in 3T3 cells determine the corresponding biological activities. Over-oxidation, a redox state more reductive than the baseline of 3T3/wt (Rb^+/+^) cells; over-reduction, a redox state more oxidative than the level in 3T3/Rb^−/−^ cells. Over-oxidative and reductive states are beyond the regulation ability of Rb and finally induce cell death. Rb regulates cell survival or transformation by controlling redox homeostasis.

To investigate whether Rb also controls redox homeostasis and transformation in human cells, we used human mammary epithelial cell line, MCF-10A, as the model. We used a lentiviral construct to knockdown Rb (shRb) [12] or over-expressed Ras^V12^ to transform human MCF-10A mammary epithelial cells [36] (Fig. 9A). Our results showed that both shRb and Ras^V12^ expression induced significant ROS generation, and that combination of Rb knockdown with Ras^V12^ expression further elevated ROS level (Fig. 9B). The oxidative state induced by Rb knockdown and/or Ras^V12^ expression was further confirmed by the ratio changes of 400/480 nm from Grx1-roGFP2 (Fig. 9C). Furthermore, we tested the colony formation ability of these cell lines in soft agar. As expected, MCF-10A/control cells did not grow in soft agar, whereas MCF-10A/Ras^V12^ cells formed well colonies in soft agar (Fig. 9D). Unlike 3T3/Rb^−/−^ cells, MCF-10A/shRb cells were able to grow in soft agar, probably due to the incomplete inactivation of Rb by knockdown (Fig. 9A). The residual functional Rb possibly still exerted its regulation on redox homeostasis to avoid an over-oxidative state that Rb knockout led to. This speculation was supported by the following results. Ras^V12^ expression in MCF-10A/shRb cells largely inhibited their growth in soft agar (Fig. 9D), because it exacerbated the oxidative redox state in MCF-10A/shRb cells (Fig. 9B and 9C). Ras^V12^ expression induced a serious oxidative state (over-oxidative redox state) in both MCF-10A/shScr and MCF-10A/shRb cells, particularly in MCF-10A/shRb cells, the addition of NAC reduced ROS level in MCF-10A cells expressing shRb and/or Ras^V12^ (Fig. 9B) as well as decreased oxidative states in all the cell lines (Fig. 9C). Accordingly, NAC dramatically increased colony growth of both MCF-10A/shRb+Ras^V12^ and MCF-10A/Ras^V12^ cells. In contrast, although Rb knockdown promoted a relative oxidative sate in MCF-10A cells (Fig. 9B and 9C), the redox state did not reach the optimal state that transformed cells at best. Therefore, NAC showed an inhibitory effect on colony growth of MCF-10A/shRb cells in soft agar (Fig. 9D). These data suggest that Rb also regulates the transformation of MCF-10A cells by controlling redox homeostasis to some proper state (Fig. 10).

## Discussion

Rb is a tumor suppressor, and regulates various biological progresses, such as cell proliferation, development, metabolism and cell death [1]–[3]. In the current study, our results show that Rb plays an important role in controlling redox homeostasis. Rb deletion in mouse 3T3 cells leads to oxidative redox and low MMP. In the meantime, Rb is demonstrated to regulate the expression of some BH3-only proteins, including Bad [30]. Bad can form an inactivating dimer with Bcl-XL and block the role of Bcl-XL in maintaining MMP [31], and its protein level is increased in 3T3/Rb^−/−^ cells. Re-expression of Rb in 3T3/Rb^−/−^ cells reduces the level of Bad, increases MMP and restores redox homeostasis. Since the expression mitochondria-targeted catalase and Bcl-XL also helps to maintain the redox homeostasis and MMP in 3T3/Rb^−/−^ cells, redox stress appears to result from mitochondria. Antioxidant NAC that blocks ROS also enhances MMP, suggesting that the elevated level of ROS can exacerbates the loss in MMP. A small amount of ROS from mitochondria can stimulate ROS generation in cytoplasm, in turn, cytoplasmic ROS can target mitochondria to release more ROS [37]–[39]. Therefore, Rb regulates redox homeostasis possibly mainly by modulating mitochondrial activity.

Rb controls cell cycle and proliferation, and it is also required for Ras^V12^-mediated transformation in 3T3 cells [34], which is confirmed by our current results. However, our results show that these functions of Rb are highly associated with Rb-controlled redox homeostasis. Rb deletion promotes the entry to S phase of cell cycle and growth of 3T3 cells, which is blocked by ROS scavenger enzyme. Transformation of 3T3 cells by Ras^V12^ also depends on the redox state regulated by Rb status or NAC.

3T3/Rb^−/−^ cells are more sensitive to oxidative stress but more tolerant towards NAC treatment in contrast to 3T3/wt cells that are sensitive to NAC treatment but not oxidative induction. At the same time, we observe that the addition of NAC leads to more reductive redox while H_2_O_2_ induces more oxidative redox in both 3T3/wt and 3T3/Rb^−/−^ cells compared to the control treatments. NAC only decreases ROS level in 3T3/Rb^−/−^ cells but leaves ROS level unaffected in 3T3/wt cells that almost do not have basal ROS. These data suggest that it is redox state but not ROS level that determines the fate of 3T3 cells and it appears that only the 3T3 cells with a proper redox state can survive (Fig. 10). The basal redox homeostasis in 3T3/Rb^−/−^ cells is more oxidative in contrast to that in 3T3/wt cells, and this difference leads to their distinct sensitivity to NAC and H_2_O_2_ treatments.

Rb deletion is able to confer the transformation ability in 3T3 cells given the redox state controlled by NAC or GSR over-expression. This suggests that Rb inactivation has the potential to transform 3T3 cells but such ability is blocked by the over-oxidative redox state. Indeed, Rb knockdown in MCF-10A cells induces oxidative redox state and transforms these cells. This inconsistency possibly results from the fact that Rb knockdown is unable to completely block Rb expression like Rb knockout and thus does not induce an over-oxidative state in MCF-10A cells. Ras^V12^ further increases the oxidative redox state in MCF-10A cells, and thus it contrarily inhibits the transformation of MCF-10A/shRb cells although it transforms MCF-10A/wt cells. These observations also support another possibility that cells with some proper redox state can be transformed while Rb is just a regulator of redox homeostasis (Fig. 10). Indeed, cancer cells usually have a more oxidative state than the normal cells. Based on this hypothesis, some sustained oxidative redox state is enough to promote cell transformation. The speculation is supported by our results that expression of Gpx1 in 3T3/wt cells increases its colony growth in soft agar, and the report that the forced expression of Mox1, a superoxide-generating oxidase, transforms the epithelial cells [40].

Over-oxidative or over-reductive redox state leads to death in 3T3 cells. 3T3/Rb^−/−^ cells have a more oxidative redox state and grows faster compared to 3T3/wt cells, but both do not grow in soft agar. However, increasing oxidative state in 3T3/wt cells or decreasing oxidative state in 3T3/Rb^−/−^ cells transform both of cell lines. These observations support the idea that different redox states in 3T3 cells determine the corresponding biological activities (Fig. 10). Cells with different redox states may undergo cell death (over-oxidation or over-reduction), survival (Rb-controlled redox), or transformation (some proper redox). Rb regulates cell survival and transformation by controlling cellular redox homeostasis. Therefore, our results suggest that Rb may be required for the initiation of some tumors, and potentially explain why Rb inactivation is restricted to a subset of human tumor cells [41]. As for those tumor cells with inactivated Rb, they may have the alternative redox regulator to allow their transformation, or acquire Rb inactivation in the progression of tumors. The underlying mechanisms remain to be explored.

ROS is involved in diverse biological progresses and tightly associated with redox homeostasis [42]. However, ROS changes are not always consistent with redox variations. For example, as for glutathione system, expression of Gpx1 or GSR show no or reduced effect on the level of ROS, but they induce respectively oxidative and reductive redox state, as detected by Grx1-roGFP. Our results show that it is redox state not ROS level is critical to determine the fate of cells. Considering that maintaining of glutathione redox balance requires ROS, the level of ROS is very important to its biological functions. Like Rb, ROS possibly exerts its biological functions by modulating the redox homeostasis. This may explain the diverse roles of ROS in biological actions.

Our results suggest that critical regulators involved in maintaining redox homeostasis could be selective targets for cancer cells. It will be of interest to further investigate the generality that redox homeostasis determines the fate of cells and the underlying mechanism. Since the redox homeostasis in cells is maintained by many components, such as glutathione system and thioredoxin system, the development of sensitive biosensors to detect in real-time the redox potential in cells is strongly desired. The quantitative analysis between redox potential and the biological activities of cells may reveal some aspect of the nature of life.

## References

[1] Dick FA, Rubin SM (2013) Molecular mechanisms underlying RB protein function. Nat Rev Mol Cell Biol 14: 297–306. 23594950 10.1038/nrm3567 PMC4754300

[2] Indovina P, Marcelli E, Casini N, Rizzo V, Giordano A (2013) Emerging roles of RB family: new defense mechanisms against tumor progression. J Cell Physiol 228: 525–535. 22886479 10.1002/jcp.24170

[3] Clem BF, Chesney J (2012) Molecular pathways: regulation of metabolism by RB. Clin Cancer Res 18: 6096–6100. 23154086 10.1158/1078-0432.CCR-11-3164

[4] Blanchet E, Annicotte JS, Lagarrigue S, Aguilar V, Clape C, et al (2011) E2F transcription factor-1 regulates oxidative metabolism. Nat Cell Biol 13: 1146–1152. 21841792 10.1038/ncb2309 PMC3849758

[5] Manning AL, Dyson NJ (2012) RB: mitotic implications of a tumour suppressor. Nat Rev Cancer 12: 220–226. 22318235 10.1038/nrc3216 PMC3800194

[6] Wise DR, Thompson CB (2010) Glutamine addiction: a new therapeutic target in cancer. Trends Biochem Sci 35: 427–433. 20570523 10.1016/j.tibs.2010.05.003 PMC2917518

[7] Wang JB, Erickson JW, Fuji R, Ramachandran S, Gao P, et al (2010) Targeting mitochondrial glutaminase activity inhibits oncogenic transformation. Cancer Cell 18: 207–219. 20832749 10.1016/j.ccr.2010.08.009 PMC3078749

[8] Vander Heiden MG, Cantley LC, Thompson CB (2009) Understanding the Warburg effect: the metabolic requirements of cell proliferation. Science 324: 1029–1033. 19460998 10.1126/science.1160809 PMC2849637

[9] Warburg O (1956) On the origin of cancer cells. Science 123: 309–314. 13298683 10.1126/science.123.3191.309

[10] Nicolay BN, Gameiro PA, Tschop K, Korenjak M, Heilmann AM, et al (2013) Loss of RBF1 changes glutamine catabolism. Genes Dev 27: 182–196. 23322302 10.1101/gad.206227.112 PMC3566311

[11] Tanaka H, Matsumura I, Ezoe S, Satoh Y, Sakamaki T, et al (2002) E2F1 and c-Myc potentiate apoptosis through inhibition of NF-kappaB activity that facilitates MnSOD-mediated ROS elimination. Mol Cell 9: 1017–1029. 12049738 10.1016/s1097-2765(02)00522-1

[12] Li B, Gordon GM, Du CH, Xu J, Du W (2010) Specific killing of Rb mutant cancer cells by inactivating TSC2. Cancer Cell 17: 469–480. 20478529 10.1016/j.ccr.2010.03.019 PMC2873973

[13] Macleod KF (2008) The role of the RB tumour suppressor pathway in oxidative stress responses in the haematopoietic system. Nat Rev Cancer 8: 769–781. 18800074 10.1038/nrc2504 PMC2989879

[14] Hanahan D, Weinberg RA (2000) The hallmarks of cancer. Cell 100: 57–70. 10647931 10.1016/s0092-8674(00)81683-9

[15] Lynch TJ, Bell DW, Sordella R, Gurubhagavatula S, Okimoto RA, et al (2004) Activating mutations in the epidermal growth factor receptor underlying responsiveness of non-small-cell lung cancer to gefitinib. N Engl J Med 350: 2129–2139. 15118073 10.1056/NEJMoa040938

[16] Mulloy R, Ferrand A, Kim Y, Sordella R, Bell DW, et al (2007) Epidermal growth factor receptor mutants from human lung cancers exhibit enhanced catalytic activity and increased sensitivity to gefitinib. Cancer Res 67: 2325–2330. 17332364 10.1158/0008-5472.CAN-06-4293

[17] Paez JG, Janne PA, Lee JC, Tracy S, Greulich H, et al (2004) EGFR mutations in lung cancer: correlation with clinical response to gefitinib therapy. Science 304: 1497–1500. 15118125 10.1126/science.1099314

[18] Kontzias A, Kotlyar A, Laurence A, Changelian P, O’Shea JJ (2012) Jakinibs: a new class of kinase inhibitors in cancer and autoimmune disease. Curr Opin Pharmacol 12: 464–470. 22819198 10.1016/j.coph.2012.06.008 PMC3419278

[19] Engelman JA, Janne PA (2008) Mechanisms of acquired resistance to epidermal growth factor receptor tyrosine kinase inhibitors in non-small cell lung cancer. Clin Cancer Res 14: 2895–2899. 18483355 10.1158/1078-0432.CCR-07-2248

[20] Rebucci M, Michiels C (2013) Molecular aspects of cancer cell resistance to chemotherapy. Biochem Pharmacol 85: 1219–1226. 23435357 10.1016/j.bcp.2013.02.017

[21] Robinson JP, Carter WO, Narayanan PK (1994) Oxidative product formation analysis by flow cytometry. Methods in Cell Biology 41: 437–447. 7861974 10.1016/s0091-679x(08)61733-1

[22] Vanden Hoek TL, Li C, Shao Z, Schumacker PT, Becker LB (1997) Significant levels of oxidants are generated by isolated cardiomyocytes during ischemia prior to reperfusion. J Mol Cell Cardiol 29: 2571–2583. 9299379 10.1006/jmcc.1997.0497

[23] Huang RF, Huang SM, Lin BS, Hung CY, Lu HT (2002) N-Acetylcysteine, vitamin C and vitamin E diminish homocysteine thiolactone-induced apoptosis in human promyeloid HL-60 cells. Journal of Nutrition 132: 2151–2156. 12163654 10.1093/jn/132.8.2151

[24] Salvioli S, Ardizzoni A, Franceschi C, Cossarizza A (1997) JC-1, but not DiOC6(3) or rhodamine 123, is a reliable fluorescent probe to assess delta psi changes in intact cells: implications for studies on mitochondrial functionality during apoptosis. FEBS Lett 411: 77–82. 9247146 10.1016/s0014-5793(97)00669-8

[25] Cossarizza A, Salvioli S (2001) Flow cytometric analysis of mitochondrial membrane potential using JC-1. Curr Protoc Cytom Chapter 9: Unit 9 14. 10.1002/0471142956.cy0914s13 18770751

[26] Gutscher M, Pauleau AL, Marty L, Brach T, Wabnitz GH, et al (2008) Real-time imaging of the intracellular glutathione redox potential. Nat Methods 5: 553–559. 18469822 10.1038/nmeth.1212

[27] Dooley CT, Dore TM, Hanson GT, Jackson WC, Remington SJ, et al (2004) Imaging dynamic redox changes in mammalian cells with green fluorescent protein indicators. J Biol Chem 279: 22284–22293. 14985369 10.1074/jbc.M312847200

[28] al Yacoub N, Romanowska M, Haritonova N, Foerster J (2007) Optimized production and concentration of lentiviral vectors containing large inserts. J Gene Med 9: 579–584. 17533614 10.1002/jgm.1052

[29] Hanson GT, Aggeler R, Oglesbee D, Cannon M, Capaldi RA, et al (2004) Investigating mitochondrial redox potential with redox-sensitive green fluorescent protein indicators. J Biol Chem 279: 13044–13053. 14722062 10.1074/jbc.M312846200

[30] Hershko T, Ginsberg D (2004) Up-regulation of Bcl-2 homology 3 (BH3)-only proteins by E2F1 mediates apoptosis. J Biol Chem 279: 8627–8634. 14684737 10.1074/jbc.M312866200

[31] Yang E, Zha J, Jockel J, Boise LH, Thompson CB, et al (1995) Bad, a heterodimeric partner for Bcl-XL and Bcl-2, displaces Bax and promotes cell death. Cell 80: 285–291. 7834748 10.1016/0092-8674(95)90411-5

[32] Verbon EH, Post JA, Boonstra J (2012) The influence of reactive oxygen species on cell cycle progression in mammalian cells. Gene 511: 1–6. 22981713 10.1016/j.gene.2012.08.038

[33] Irani K, Xia Y, Zweier JL, Sollott SJ, Der CJ, et al (1997) Mitogenic signaling mediated by oxidants in Ras-transformed fibroblasts. Science 275: 1649–1652. 9054359 10.1126/science.275.5306.1649

[34] Williams JP, Stewart T, Li B, Mulloy R, Dimova D, et al (2006) The retinoblastoma protein is required for Ras-induced oncogenic transformation. Mol Cell Biol 26: 1170–1182. 16449633 10.1128/MCB.26.4.1170-1182.2006 PMC1367176

[35] Ho E, Chen G, Bray TM (1999) Supplementation of N-acetylcysteine inhibits NFkappaB activation and protects against alloxan-induced diabetes in CD-1 mice. FASEB J 13: 1845–1854. 10506589

[36] Premzl A, Puizdar V, Zavasnik-Bergant V, Kopitar-Jerala N, Lah TT, et al (2001) Invasion of ras-transformed breast epithelial cells depends on the proteolytic activity of cysteine and aspartic proteinases. Biol Chem 382: 853–857. 11517941 10.1515/BC.2001.104

[37] Zhang DX, Gutterman DD (2007) Mitochondrial reactive oxygen species-mediated signaling in endothelial cells. Am J Physiol Heart Circ Physiol 292: H2023–2031. 17237240 10.1152/ajpheart.01283.2006

[38] Coughlan MT, Thorburn DR, Penfold SA, Laskowski A, Harcourt BE, et al (2009) RAGE-induced cytosolic ROS promote mitochondrial superoxide generation in diabetes. J Am Soc Nephrol 20: 742–752. 19158353 10.1681/ASN.2008050514 PMC2663823

[39] Zorov DB, Juhaszova M, Sollott SJ (2006) Mitochondrial ROS-induced ROS release: an update and review. Biochim Biophys Acta 1757: 509–517. 16829228 10.1016/j.bbabio.2006.04.029

[40] Suh YA, Arnold RS, Lassegue B, Shi J, Xu X, et al (1999) Cell transformation by the superoxide-generating oxidase Mox1. Nature 401: 79–82. 10485709 10.1038/43459

[41] Horowitz JM, Park SH, Bogenmann E, Cheng JC, Yandell DW, et al (1990) Frequent inactivation of the retinoblastoma anti-oncogene is restricted to a subset of human tumor cells. Proc Natl Acad Sci U S A 87: 2775–2779. 2181449 10.1073/pnas.87.7.2775 PMC53773

[42] Circu ML, Aw TY (2010) Reactive oxygen species, cellular redox systems, and apoptosis. Free Radic Biol Med 48: 749–762. 20045723 10.1016/j.freeradbiomed.2009.12.022 PMC2823977

